# High expression level of miR-1260 family in the peripheral blood of patients with ovarian carcinoma

**DOI:** 10.1186/s13048-021-00878-x

**Published:** 2021-10-10

**Authors:** Arash Adamnejad Ghafour, Demet Akdeniz Odemis, Seref Bugra Tuncer, Busra Kurt, Mukaddes Avsar Saral, Seda Kilic Erciyas, Ozge Sukruoglu Erdogan, Betul Celik, Pinar Saip, Hulya Yazici

**Affiliations:** 1 Division of Cancer Genetics, Department of Basic Oncology, Oncology Institute, Istanbul University, Fatih, 34093 Istanbul, Turkey; 2 Health Science Institute, Istanbul University, Fatih, 34093 Istanbul, Turkey; 3 Health Services Vocational School of Higher Education, T.C. Istanbul Aydin University, Sefakoy, Kucukcekmece, 34295 Istanbul, Turkey; 4 Department of Medical Oncology, Oncology Institute, Istanbul University, Fatih, 34093 Istanbul, Turkey; 5 Medical Biology and Genetics Department, Medical Faculty, Istanbul Arel University, Istanbul, Zeytiburnu 34010 Turkey

**Keywords:** Familial and Sporadic Ovarian Cancer, miRNA expression, Biomarker

## Abstract

The most common gynecologic cancers detected in women in Turkey are uterine cancer, ovarian cancer, and cervical cancer. These data reported that a mean of 3800 individuals were diagnosed with uterine cancer, 2790 were diagnosed with ovarian cancer, and 1950 were diagnosed with cervical cancer, and 400 individuals were diagnosed with other gynecologic cancers each year in Turkey. A mean of 14.270 individuals were detected to have been diagnosed with gynecologic cancers each year in the United States of America (USA). Ovarian cancer treatment is generally composed of chemotherapy, and surgery. In general, chemotherapy is administered after surgery. The identification of the molecular pathogenesis of ovarian cancer, and discovery of new moleculer biomarkers which facilitate the ovarian cancer treatment are required for an effective ovarian cancer treatment in clinics. miRNAs are reported to be the possible biologic indicators for various cancer types. We aimed to investigate 2 miRNAs which were suggested to have effect in ovarian cancer in our (previous) monozygotic twin study from miR-1260 microRNA family whose association with ovarian cancer yet has not been reported in the literature. We investigated the expression levels of miR-1260a, and miR-1260b miRNAs, in the peripheral blood lymphocytes of 150 familial and sporadic ovarian cancer patients, and of 100 healthy individuals of the control group who were matched for age, sex, and ethnicity with the patient group, and investigated their possible property of being a biologic indicator for ovarian cancer. The expression results of ovarian cancer patients were evaluated by comparison of the results of the control group in the study. The expression levels of miR-1260a, and miR-1260b in ovarian cancer patients were found highly increased compared with the levels in the control group. miR-1260a expression level in ovarian cancer patients was detected to have increased approximately 17 fold compared with the control group, and miR-1260b expression level in ovarian cancer patients was detected to have increased approximately 33 fold compared with the levels in the control group. The String Analyses showed that the miR-1260a was associated with the ribosomal protein family which was known to be effective in the translation stage of cell and that miR-1260b was associated with *CHEK2* protein which was a member of the serine/threonine-protein kinase family. It should be investigated for larger cohorts in benign ovarian diseases and in different stages of patients receiving ovarian cancer treatment whether these two molecules are a noninvasive biomarker and therapeutic target to be used especially in the early diagnosis and prognosis of ovarian cancer in future.

## Introduction

Approximately 22.240 individuals were diagnosed with ovarian cancer in the USA in 2018, and 14.070 were reported to have died of ovarian cancer [1]. Malignant tumors constitute 2.5% of gynecologic tumors in women. 5% of women diagnosed with these cancers died of advanced stage disease [2, 3]. Age-adjusted incidence rates have been observed in rates generally higher than 8 in 100.000 in the developed regions of the world including the North America, Central, and East Europe. The rates in South America was 5.8 in 100.000, and the mean rate was 3 in 100.000 in Asia, and Africa [4, 5]. Ovarian cancer biology is different than the hematogenously metastating tumors because the ovarian cancer cells are invased into the peritoneal cavity. Ovarian carcinoma with a treatment rate of 30% is a fatal disease owing to the compression to the visceral organs of rapidly proliferation of tumors, and to the low chemosensitivity of the cells. Ovarian cancer tumorogenesis proceeds through a gradual mutation period from a gradually growing borderline tumor to a well-differentiated carcinoma (Type I) or through a genetically nonstable high degree serous carcinoma with rapid metastasis (Type II) [3].

Two different molecular sub-classes have recently been differentiated using the genetic changes in different histologic subtypes of ovarian cancer. The first category consists of mucinous borderline ovarian tumors with the potential of low degree serous papillary, endometrioid, and low malignity (type I cancers), and the second category consists of high degree serous carcinomas (type II cancers) [6, 7]. The most common subtype of epithelial ovarian carcinoma of the high-grade serous carcinoma (HGSC) is a different disease than the low grade serous carcinoma (LGSC). HGSC generally develops in advanced age, and the response to chemotherapy is good, however the general prognosis is poorer compared than in the LGSC. On the contrary, LGSC developes in the younger ages, and the prognosis is better [8–11]. Ovarian carcinoma is a disease generated by a series of genetic, and epigenetic changes that cause cell transformation [12]. The meta-analyses showed that the risk of developing ovarian cancer in patients with *BRCA1* mutation was cumulatively higher. Accordingly, the cumulative risk of ovarian cancer until the age of 70 was 39-40% in patients with BRCA1 mutation, and this risk was reported as a mean of 11-70% in patients with *BRCA2* mutation [13].

microRNAs (miRNA) are the noncoding RNAs that were first discovered in 1993 in Caenorbabditis Elegans [14, 15]. miRNA molecules were also reported to be found in all plant, and animal types [16]. More than 50% of miRNA genes are known to have presented as multicistronic RNA transcripts, and some were located intragenically in a different gene named as the host gene [17]. Approximately 40% of intergenic miRNAs are located in the intronic regions of the noncoding transcripts, and 10% are located in the exonic regions [18, 19].

miRNAs were shown to cause functional changes in cancer cells, and therefore had roles in various cancers [20]. miR-1260a was shown to be located on 14q24.3 chromosome region, and this miRNA was upregulated in breast cancer, malignant melanoma, hepatocellular carcinoma, colorectal carcinoma, esophageal squamous cell carcinoma, kidney carcinoma, and gastric cancer [21–28]. Although there was no study in the literature suggesting that miR-1260a was specifically associated with ovarian cancer, miR-1260a was suggested to have possibly been upregulated in ovarian cancer in the assumption after the results of the study investigating that in prostate cancer patients [27, 29]. In addition, the higher expression level of miR1260a, and miR-1260b was also reported in the serum, and FFPE tissues of prostate cancer (PC) patients, and in prostate cell series [29–31]. A new kit was developed, and used which enabled the investigation in the FFPE tissues of patients for diagnosis and molecular classification of prostate cancer patients in Denmark population with the results of cancer associated miRNA studies [29]. miR-1260b was shown to have been expressed in abnormal levels in various tumors, and in human dendritic cells, and miR-1260b was suggested to have a possible role in the immune reaction of dendritic cells in the literature [32]. The anti-tumor effect of Genistein in prostate cancer cell strains was reported to be performed by the downregulation of miR-1260b which targeted the *SRRP1*, and *SMAD4* genes [33]. miR-1260b expression level was reported to have increased in colorectal carcinomas (CRC), and was upregulated particularly in the infiltrative lymph nodes of CRC patients, and higher miR-1260b expression was shown to be associated with lymph node metastasis, and venous invasion [34]. These results showed that miR-1260b might identify the the early period CRC metastasis, and was associated with poor prognosis, and therefore miR-1260b was suggested to be a possible significant molecular bioindicator in CRC prognosis [34].

We investigated the expression levels of miR-1260a, and miR-1260b in the peripheral blood lymphocytes of ovarian cancer patients, and healthy controls in the present study in accordance with the evaluations recently suggesting that miRNAs had biologic indicator, and therapeutic target properties, and due to the lack of a sensitive and noninvasive biologic indicator for utilization in the early diagnosis, treatment and, follow-up of ovarian cancer, and these two molecules were evaluated whether they had noninvasive early biologic indicator properties for ovarian cancer.

## Material and method

### Study groups

The study was approved by the Ethics Board of Istanbul University, Istanbul Faculty of Medicine (Ethics Board approval dated 28.05.2018, and with the ethics board decision no: 01). The peripheral blood samples taken from 150 ovarian cancer patients who presented to Cancer Genetics clinic in Istanbul  University, Oncology Institute between 2012, and 2017, and who confirmed the use of the residual material of the routine test for research purpose, and from 100 healthy controls who were matched for age, sex, and ethnicity with the patient group, and with no history of cancer in 3 generation relatives in their family, were used. The blood was drawn after the informed consent form was signed by all participants included in the study. The study was pursued with the materials of 127 patients, and 98 healthy individuals after the quality analyses were performed.

### Lymphocyte isolation from peripheral blood

The peripheral blood lymphocytes were differentiated from other blood components using the Ficoll method. The blood sample diluted in a ratio of 1:1 (v/v) with Phosphate Buffer Solution (PBS) was loaded on 2 mL Ficoll-Histopaque-1077 (Sigma-Aldrich, USA) using a pipette, and centrifugated for 30 min at room temperature at 400Xg. The differentiated lymphocytes in the medium phase of the tube after centrifugation procedure were transferred into a clean tube using a pasteur pipette. The differentiated cells were precipitated by centrifugating at room temperature at 400Xg for 10 min. The supernatant was removed, and 2 mL PBs was added on the precipitated pellete, and pellete was thawed by pipetting. The thawed cells were distributed in 2 cryotubes 1 mL in each tube, and then centrifugated at room temperature at 400Xg for 4 min. Supernantants were removed. The cells precipitated in cryotube were incubated for 1 day at -80°C. Then, the cells in the cryotubes were placed in liquid nitrogen storage tank for long term storage.

### RNA isolation

A commercial Quick-RNA MiniPrep Kit [Zymo Research, CA, USA ] protocol was used for isolating RNA from lymphocytes. 300 μL RNA Lysis Buffer was added on lymphocytes in accordance with the kit protocol, and was centrifugated at +4°C at 12.000Xg for 30 sec. The supernatant was tranferred into yellow column collection tubes (Spin Away Filter), and 300 μL 95-100 % ethanol was added, and the mixture was centrifugated for 30 sec at +4°C at 10.000Xg. The phase at the bottom was transferred into green column collection tubes (Zymo-Spin IIICG Column). The preparate was incubated at room temperature for 15 min after adding 5 μL DNase I, and 75 μL DNA Digestion Buffer. 400 μL RNA Prep Buffer was added on the column, and was centrifugated at +4°C at 12.000Xg for 30 sec. The bottom phase was removed. 700 μL RNA Wash Buffer was added on the column, the bottom phase was removed again after centrifugation for 30 sec at +4°C at 12.000Xg. 400 μL RNA Wash Buffer was added on the column for repeating the washing procedure, and was centrifugated for 2 min. at +4°C at 12.000Xg. The RNAse noninvolving column was tranferred into a clean centrifugating tube of 1.5 mL, and 100 μL sterilised distilled water with no inclusion of DNAse/RNAse was added, and centrifugated at +4°C at 12.000Xg for 30 seconds. The obtained RNAs were stored at -80^°^C in several different tubes after quality controls were performed.

### Identification of the quality control, and concentrations of RNA samples

The quantity analyses, and quality controls of the obtained RNAs were performed using a Nanodrop 2000 Spectrophotometer [Thermo Fisher Scientific, USA]. The purity, and concentration measurement of the extracted RNA was performed by reading the absorbance at A260/A280 nm wave-lengths after the direct loading of the 1μL stock RNA sample. The RNAs with the absorbance between 1.6-2.0 OD were accepted as appropriate for expreimental procedures.

### cDNA synthesis

RNA samples were dissolved, and then vortexed. RNA with a maximum volume of 1 μg templeta RNA, 5 μL ID3AL RT Buffer, 1 μL ID3AL RT Primer, and 1 μL ID3AL RT (Reverse Transcriptase) were respectively added in the tube for cDNA reaction. The volume of the obtained mixture was completed to 20 μL with distilled water. Pipetaging was performed using a micropipete, and then vortexed. The cDNA forming procedure was completed using a BioRad PCR device with incubation for 30 min at 42°C, and for 5 min at 95°C.

### Real time qPCR reaction, and measurement of CT value

The obtained cDNAs were diluted using sterilised DNA/RNAse free distilled water with a ratio of 1:10(v:v). The diluted cDNAs were stored at -20°C. 5 μL diluted cDNA, 10 μL ID3AL qPCR Master Mix, 2 μL ID3AL qPCR assays, and 3 μL distilled water were added to obtain a total volume of 20 μL for real time qPCR reaction Pipetaging was performed using a micropipete, and inserted on Mic qPCR Cycler (Bio Molecular Systems, Australia) device after vortexing. All qPCR reactions were performed in duplicated manner. The quantitative PCR reactions were performed in duplicated manner. The quantitative PCR procedure was performed in 40 cycles with incubation at 95°C for 10 min, and then at 40°C for 5 min, then at 95°C for 40 sec, and at 60°C for 30 sec. The measurement of the reactions, and calculations were performed using the Mic qPCR software on the device. The miRNA expression levels investigated with this software were calculated quantitatively using the below formula [35].

$$\Delta \mathrm{CT}=\mathrm{CT}\ \left(\mathrm{Target}\ \mathrm{Gene}\right)-\mathrm{CT}\ \left(\mathrm{Reference}\ \mathrm{Gene}\right).$$

$$\Delta \Delta \mathrm{CT}=\Delta \mathrm{CT}\ \left(\mathrm{Target}\ \mathrm{Gene}\right)-\sum \Delta \mathrm{CT}\ \left(\mathrm{Reference}\ \mathrm{Gene}\right).$$

$$\mathrm{As}\ \Delta \Delta \mathrm{Ct}\ \mathrm{value}\ \mathrm{of}\ 0,\mathrm{and}\ {2}^0=1,\mathrm{the}\ \mathrm{reference}\ \mathrm{value}\ \mathrm{was}\ \mathrm{taken}\ \mathrm{as}\ 1.$$

### Statistical analyses

The associations, and evaluations of the miRNA-1260 expression levels of patients, and healthy controls, and the clinical parameters were performed using the χ^2^ tests (Yates, Fisher’s exact) SPSS.21 statistical program, ANOVA test, and String Analysis. The Kaplan-Meier Analysis was used for identifying whether the expression levels of miR-1260a, and miR-1260b were tha variables affecting the survival; and the ROC analysis was performed for demonstrating the diagnostic performance of the candidate bioindicators miR-1260a, and miR-1260b molecules. The Spearman’s correlation test was used for identifying the possible linear association between Ca-125 level of ovarian cancer patients, and the miRNA expression levels.

## Results

The samples of 150 patients diagnosed with ovarian cancer, and 100 healthy controls were used in the study. The study was pursued with 127 patients, and 98 healthy controls after quality control analysis. The mean age of the ovarian cancer patient group was 50±10 years (25-84y), and the mean age of the control group was 48±11years (23-84y). 103 ovarian cancer patients were only diagnosed with ovarian cancer, 16 were diagnosed with an additional breast cancer, 2 were diagnosed with an additional endometrium cancer, and 6 were diagnosed with a different secondary cancer than the cancers described above.

The normality estimation of the groups which was evaluated within the scope of the research using the SPSS v21.0 program was performed using the Kolmogorov-Smirnov test. The normality distribution of the data for miR-1260a was (Kolmogorov-Smirnov Z=5,274; p<0.05); and for miR-1260b (Kolmogorov-Smirnov Z=5,361; p<0.05. We used the post-hoc ANOVA test in the resolution of the data because the groups showed a normal distribution, and were investigated in 5 groups. The calculations performed based on the 2^-∆∆Ct^ values of the patient, and control groups with the evaluations using the ANOVA test showed that the miR-1260a, and miR-1260b expression levels were statistically significant in patient groups compared with the levels in the control groups (p:0.000). We performed the Post-Hoc Tukey HSD test for detailed demonstration of the significance between the subgroups. The results of this test showed that there was a statistically significant difference for both miRNAs between the healthy controls, and in the patient groups with ovarian cancer, ovarian and breast cancer, ovarian and endometrium cancer, and ovarian and other cancer patients (p:0.000) (Table 1).

**Table 1:** ANOVA test results

Dependent Variable	Group 1	Group 2	Mean Difference	Standard Error	p Value	%95 Confidence Interval Lower limit Upper limit	
**miR-1260a**							
	**Control Group**	**All Ovarian Cancer**			.000*		
		**Ovarian + Breast Cancers**	1.37500^*^	.09612	.000*	1.1106	1.6394
		**Only Ovarian Cancer**	1.35922^*^	.05031	.000*	1.2209	1.4976
		**Ovarian + Endometrium Cancer**	1.50000^*^	.25464	.000*	.7996	2.2004
		**Ovarian + Other Cancers**	1.00000^*^	.14993	.000*	.5876	1.4124
**miR-1260b**							
	**Control Group**	**All Ovarian Cancer**			.000*		
		**Ovarian+ Breast Cancers**	125000^*^	.08968	.000*	1.0033	1.4967
		**Only Ovarian Cancer**	1.28155^*^	.04693	.000*	1.1525	1.4106
		**Ovarian + Endometrium**	1.50000^*^	.23756	.000*	.8466	2.1534
		**Ovarian + Other Cancers**	1.00000^*^	.13987	.000*	.6153	1.3847

*; statistically significant (*p*<0.05)

**Fig. 1:**
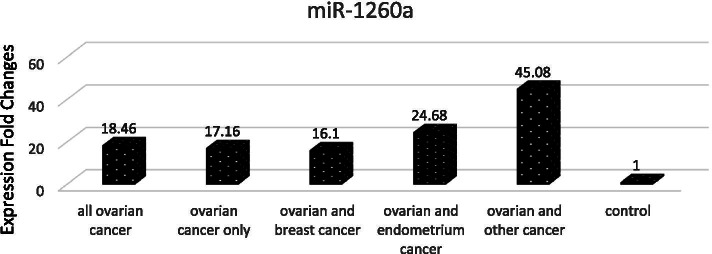
Graphic Representation of Proportional Differences of miR-1260a Expression in all Patients with Ovarian Carcinoma and Subgroups According to Healthy Controls

The comparison of the miR-1260a expression levels of the healthy control group, and ovarian cancer patients showed that in general miR-1260a expression level was 18.46 fold higher in all patients compared with the levels in healthy women. miR-1260a expression was found as 17.16 fold higher in patients diagnosed with only ovarian cancer, as 16.1 fold higher in patients with two primary cancers as ovarian and breast cancer, the values were increased in 24.68 fold in patients diagnosed with both ovarian and endometrium cancer, and as 40.08 fold in patients diagnosed with a different additional secondary cancer than above with ovarian cancer (Fig. 1).

**Fig. 2:**
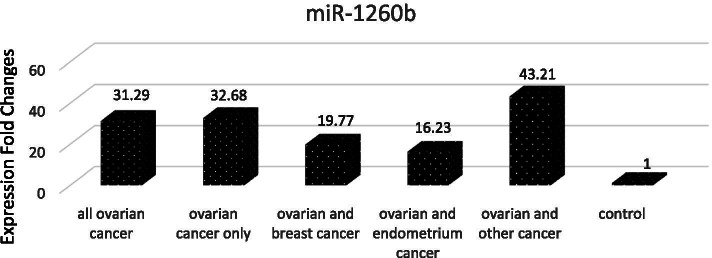
Graphic Representation of Proportional Differences of miR-1260b Expression in Total Patients with ovarian carcinoma and Subgroups According to Healthy Controls

The comparison of miR-1260b between healthy control group and ovarian cancer groups showed that in general miR-1260b expression level was 31.29 fold in all patients compared with the levels in healthy women. miR-1260b expression was found to have increased in 32.68 fold in patients with only ovarian cancer, and as 19.77 fold in patients with two primary cancers as ovarian and breast cancers, and as 16.23 fold in patients with ovarian and endometrium cancers, and 43.21 fold increase was detected in ovarian cancer patients with a different additional secondary cancer (Fig. 2).

The evaluation of all ovarian cancer patients showed that miR-1260a expression was decreased in 34.7% (44/127) of patients, and was increased in 65.3% (83/127) of patients, however, miR-1260b expression was lower in 26.8% (34/127) of patients, and was increased in 73.2% (93/127) of patients (Table 2).

**Table 2:** Distribution of miR-1260a, and miR-1260b expression levels in patients with ovarian cancer

Diagnosis	miR-1260a Expression		Total	miR-1260b Expression		Total
	Increased n(%)	Decreased n(%)	Total n (%)	Increased n(%)	Decreased n(%)	Total n (%)
**Patients with Only Ovarian Cancer**	66(64.1%)	37(35.9%)	103(81.1%)	74(71.8%)	29(28.2%)	103(81.1%)
**Patients Diagnosed With both Ovarian and Breast Cancer**	10(62.5%)	6(37.5%)	16(12.6%)	12(75.0%)	4(25.0%)	16(12.6%)
**Patients with both Ovarian and Endometrial Cancer**	1 (50.0%)	1(50.0%)	2(1.6%)	1 (50.0%)	1(50.0%)	2(1.6%)
**Patients with Ovarian and Other Cancers**	6(100.0%)	0(00.0%)	6(4.7%)	6(100.0%)	0(0.0%)	6(4.7%)
**Total n (%)**	83(65.3%)	44(34.7%)	127	93(73.2%)	34(26.8%)	127

miR-1260a expression was increased in 64.1% (66/103) of 103 patients diagnosed with only ovarian cancer out of the total of 127 ovarian cancer patients, and was decreased in 35.9% (37/103) of the patients. miR-1260a expression levels of 16 patients diagnosed with both ovarian, and breast cancer were found to have increased in 62.5% (10/16), and was found to have decreased in 37.5% (6/16) of the patients. miR-1260a expression was increased in 50%(1/2), and was decreased in 50% of patients diagnosed with different primary cancers as ovarian, and endometrium cancer. miR-1260a expression was detected to have increased in 100%(6/6) of patients diagnosed with ovarian cancer, and another secondary cancer different than the above reported cancers (Table 2).

miR-1260b expression was increased in 71.8% (74/103), and was decreased in 28.2% (29/103) in 103 patients with only ovarian cancer out of 127 ovarian cancer patients. miR-1260b expression levels were found to have increased in 75% (12/16), and was decreased in 25% (4/16) of patients with both ovarian, and breast cancer. miR-1260b expression was increased in 50%, and was decreased in 50% of patients with ovarian, and endometrium cancer. miR-1260b expression was detected to have increased in all ovarian cancer patients with an additional secondary cancer (Table 2).

### Comparison of the miR-1260a, and miR-1260b Expression Levels with the Clinical Characteristics

The investigation of the distribution of miR-1260a, and miR-1260b expression levels in different clinical stages of ovarian cancer patients showed that miR-1260a expression level was decreased in 30.5% (11/36), and was increased in 69.5%(25/36) in Stage I/II patients. miR-1260a expression level was found lower in 36.3% (33/91), and was higher in 63.7% (58/91) of ovarian cancer patients in Stage III/IV. However, miR-1260b expression level was found to have decreased in 25% (9/36), and was increased in 75% (27/36) of patients in Stage I/II, and the expression level was found lower in 27.5% (25/91), and was found to have increased in 72.5% (66/91) of patients in Stage III /IV (Table 3).

**Table 3:** Comparison of miR-1260a, and miR-1260b expression levels, and the clinical features of patients

Features	miR-1260a	Expression	miR-1260b	Expression	
Expression Level	Increased	Decreased	Increased	Decreased	Total
	n (%)	n (%)	n (%)	n (%)	n(%)
**Mean age 50 ± 10 y(25-84y)**					
**40 y>**	11 (8.7%)	5 (3.9%)	13 (10.2%)	3 (2.4%)	16 (12.5%)
**40 y≤**	72 (56.7%)	39 (30.7%)	31 (24.4%)	80 (63%)	111 (87.5%)
**Total n (%)**	83 (65.3%)	44 (34.7%)	44 (34.7%)	83 (65.3%)	127
**Clinical Stage**					
**Stage 1**	12 (9.4%)	10 (7.9%)	14 (11.0%)	8 (9.3%)	22 (17.3%)
**Stage 2**	13 (10.2%)	1 (0.8%)	13 (10.2%)	1 (0.8%)	14 (11%)
**Stage 3**	49 (38.6%)	27 (21.3%)	56 (44.1%)	20 (15.7%)	76 (59.9%)
**Stage 4**	9 (7.1%)	6 (4.7%)	10 (7.9%)	5 (%3.9)	15 (11.8%)
**Total n(%)**	83 (65.3%)	44 (34.7%)	93 (73.2%)	34(26.8%)	127
**Pathological Grade**					
**Grade 1**	11 (8.7%)	11 (8.7%)	16 (12.6%)	6 (%4.7)	22 (17.3%)
**Grade 2**	14 (11.0%)	5 (3.9%)	14 (11.0%)	5 (3.9%)	19 (15%)
**Grade 3**	58 (45.7%)	28 (22.0%)	63 (49.6%)	23 (18.1%)	86 (67.7%)
**Total n(%)**	83 (65.3%)	44 (34.6%)	93 (73.2%)	34 (26.8%)	127
**Histological Grade**					
**Grade 1**	12 (9.4%)	13 (10.2%)	16 (12.6%)	9 (7.1%)	25 (19.6%)
**Grade 2**	13 (10.2%)	11 (8.7%)	17 (13.4%)	7 (%5.5)	24 (18.9%)
**Grade 3**	58 (45.7%)	20 (15.7%)	60 (47.2%)	18 (14.2%)	78 (61.5%)
**Total n(%)**	83 (65.3%)	44 (34.6%)	93 (73.2%)	34 (26.8%)	127
**Tumor Size**					
**<2cm**	18 (14.2%)	10 (7.9%)	20 (15.7%)	8 (6.3%)	28 (22%)
**≥2cm**	65 (51.2%)	34 (26.8%)	73 (57.5%)	26 (20.5%)	99 (78%)
**Total n(%)**	83 (65.3%)	44 (34.6%)	93 (73.2%)	34 (26.8%)	127
**Treatment**					
**Surgery**					
**Yes**	78 (63.9%)	44 (32.1%)	87 (69.0%)	34 (27.0%)	122 (96%)
**No**	5 (4.0%)	0 (0.0%)	5 (4.0%)	0 (0.0%)	5 (4%)
**Total n(%)**	83 (65.3%)	44 (34.6%)	93 (73.2%)	34 (26.8%)	127
**Chemotherapy**					
**Yes**	78 (66.1%)	40 (25.9%)	86 (68.3%)	30 (23.8%)	118 (92.9%)
**No**	5 (4.0%)	5 (4.0%)	6 (4.8%)	4 (3.2%)	10 (7.1%)
**Total n(%)**	83 (65.3%)	44 (34.6%)	93 (73.2%)	34 (26.8%)	127
**Radiotherapy**					
**Yes**	10 (7.9%)	5 (4.0%)	10 (7.9%)	5 (%4.0%)	15 (11.8%)
**No**	73 (57.4%)	39 (30.7%)	82 (65.1%)	29 (23.0%)	112 (88.2%)
**Total n(%)**	83 (65.3%)	44 (34.6%)	93 (73.2%)	34 (26.8%)	127
**Ovarian & Breast cancer histories in the family**					
**<2 persons**	52 (40.9%)	31 (24.4%)	59 (46.5%)	24 (%18.8%)	83 (65.3%)
**≥2 persons**	31 (24.4%)	13 (10.3%)	34 (26.8%)	10 (7.9%)	44 (34.7%)
**Total n(%)**	83 (65.3%)	44 (34.7%)	93 (73.2%)	34 (26.8%)	127

The evaluation of the expression level of ovarian cancer patients in accordance with the pathologic grade showed that miR-1260a expression was decreased in 50% (11/22), and was increased in 50% (11/22) of patients with pathologic Grade I. The miR-1260a expression level was decreased in 26.3% (5/19), and was increased in 73.7% (14/19) in patients with pathologic grade II. miR-1260a expression level was decreased in 32.5% (28/86), and was increased in 67.5% (58/86) of patients with pathologic grade III. miR-1260b expression was decreased in 27.3% (6/22), and was increased in 72.7% (16/22) of patients with pathologic grade I. miR-1260b expression level was decreased in 26.3% (5/19), and was increased in 73.7% (14/19) patients with pathologic grade II. miR-1260b expression level was decreased in 26.7% (23/86), and was increased in 73.3% (63/86) of patients with pathologic Grade III (Table 3).

The evaluation of ovarian cancer in accordance with the histologic grade showed that miR-1260a expression level was increased in 52% (13/25), and was decreased in 48% (12/25) of patients with histologic Grade I. miR-1260a expression level was increased in 45.8% (11/24), and was decreased in 54.2% (13/24) of patients with histologic Grade II. miR-1260a expression level was increased in 25.6% (20/78), and was decreased in 74.4% (58/78) of patients with histologic Grade III. miR-1260b expression was increased in 36% (9/25), and was decreased in 64% (16/25) of patients with histologic Grade I. miR-1260b expression was increased in 29.1% (7/24), and was decreased in 70.9% (17/24) of patients with histologic Grade II. miR-1260b expression was increased in 23% (18/78), and was decreased in 77% (60/78) of patients with histologic Grade III (Table 3).

The evaluation of ovarian cancer patients in accordance with the tumor size showed that miR-1260a expression was increased in 64.3% (18/28), and was decreased in 35.7% (10/28) of patients with a tumor diameter smaller than 2 cm. miR-1260a expression level was increased in 65.7% (65/99), and was decreased in 34.3% (34/99) of patients with a tumor diameter larger than 2 cm. miR-1260b expression was increased in 71.4% (20/28), and was decreased in 28.6% (8/28) of patients with a tumor diameter smaller than 2 cm. The expression level was increased in 73.7% (73/99), and was decreased in 26.3% (26/99) of patients with a tumor diameter larger than 2 cm (Table 3).

The evaluation of ovarian cancer patients in accordance with their treatment type showed that miR-1260a expression level was increased in 63.9% (78/122), and was decreased in 36.1% (44/122) of patients who underwent surgery. miR-1260a expression level was increased in 100% (5/5), and no decrease was detected in expression levels of patients who did not undergo surgery. miR-1260b expression level was increased in 71.3% (87/122), and was decreased in 28.7% (34/122) of patients who underwent surgery. miR-1260b expression level was increased in 100% (5/5), and no decrease was detected in expression levels of patients who did not undergo surgery (Table 3).

miR-1260a expression level was increased in 66.1% (78/118), and was decreased in 33.9% (40/118) of patients who underwent chemotherapy. miR1260a expression level was found to have increased in 50%, and to have decreased in 50% of patients who received no chemotherapy. miR-1260b expression level was increased in 72.8% (86/118), and was decreased in 27.2% (30/118) of patients who underwent chemotherapy. miR-1260b expression level was increased in 60% (6/10), and was decreased in 40% (4/10) of patients who received no chemotherapy (Table 3).

miR-1260a expression level was found to have increased in 66.6% (10/15), and to have decreased in 33.4% (5/15) of patients who received radiotherapy, however, miR-1260a expression level was found to have increased in 65.1% (73/112), and to have decreased in 34.9% (39/112) of patients who received no radiotherapy. The miR-1260b expression level was found to have increased in 66.6% (10/15), and to have decreased in 33.4% (5/15) of patients who received radiotherapy however, miR-1260b expression level was found to have increased in 73.2% (82/112), and to have decreased in 26.8% (29/112) of patients who received no radiotherapy (Table 3).

### Evaluation of the association between the miR-1260a, and miR-1260b expression levels, and family history

miR-1260a expression was detected to have decreased in 37.4% (31/83), and have increased in 62.6% (52/83) of patients with ovarian cancer and/or breast cancer patients in less than two individuals, miR-1260a expression was detected to have decreased in 29.6% (13/44), and have increased in 70.4% (31/44) of patients with 2 or more than 2 ovarian cancer and/or breast cancer patients in the family. miR-1260b expression was detected to have decreased in 29% (24/83), and have increased in 71% (59/83) of patients with ovarian cancer and/or breast cancer patients in less than two individuals in the family however, miR-1260b expression was detected to have decreased in 22.8% (10/44), and have increased in 77.2% (34/44) of patients with 2 or more than 2 ovarian cancer and/or breast cancer patients in the family.

### Evaluation of the miR-1260a, and miR-1260b Expression levels with the presence of BRCA1/BRCA2 Gene Mutations

miR-1260a expression was detected to have increased in 76% (19/25), and decreased in 24% (6/25) in BRCA positive ovarian cancer patients, and have increased in 62.7% (64/102), and decreased in 37.3% (38/102) BRCA negative patients in the study.

miR-1260b expression was detected to have increased in 72% (18/25), and decreased in 28% (7/25) in BRCA positive ovarian cancer patients, and have increased in 73.5% (75/102), and decreased in 26.5% (27/102) BRCA negative patients.

### Survival analysis

The mean survival time of ovarian cancer patients in the present study was 103 months (SD ± 5.7 m), and 45(35.4%) died, 78 (61.4%) survived after diagnosis, and the situation of 4(3.2%) were unknown out of 127 patients that were screened between 2010-2019.

**Fig. 3:**
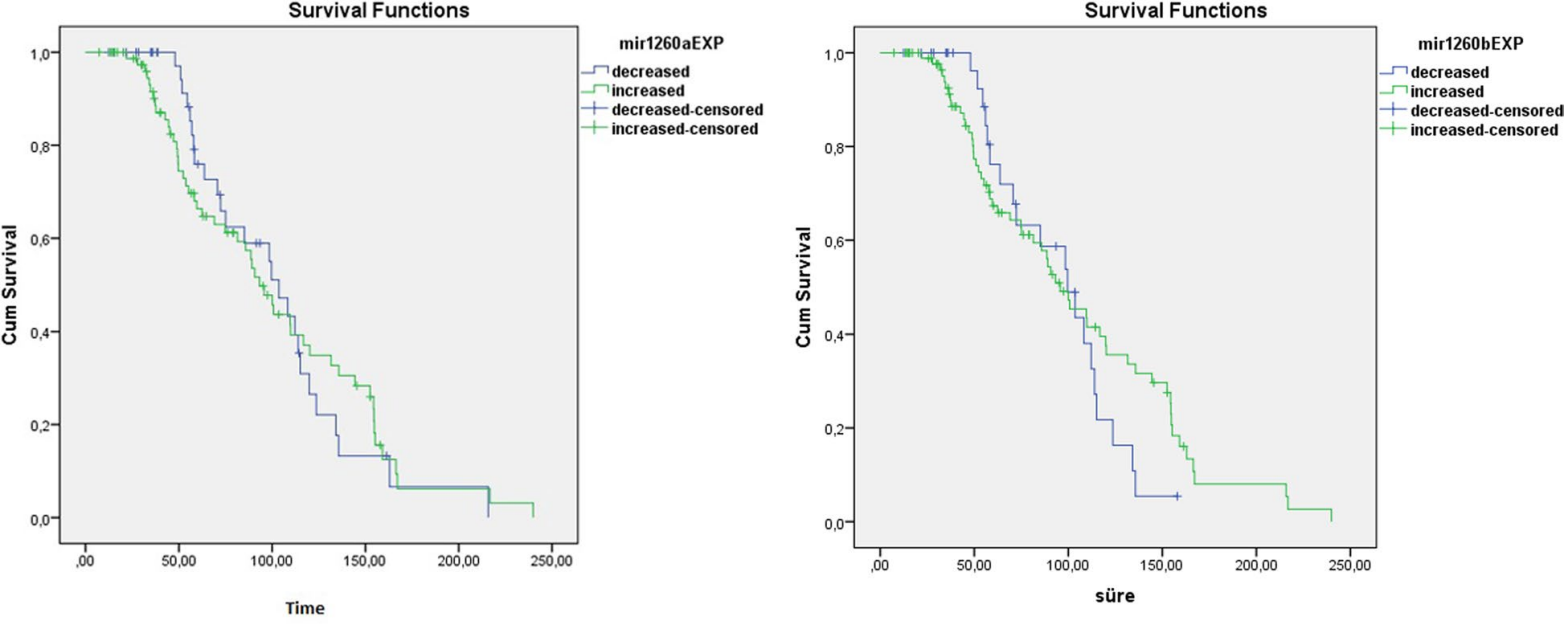
Survival Analysis for both miR-1260a, and miR-1260b

We performed the Kaplan-Meier Analysis to investigate whether miR-1260a, and miR-1260b expression levels were the variables affecting the survival time. No correlation was detected between the miR-1260a, and miR-1260b expression levels with the survival time (Table 4) (Fig. 3).

**Table 4:** Correlations Between miRNAs Expression Levels, and Survival

miR-1260a Expression	Total N	N of Events	Censored	
			N	Percent
Decreased	44	26	18	40.9%
Increased	83	52	31	37.3%
Ovarian all	127	78	49	38.6%
miR-1260b Expression				
Decreased	34	20	14	41.2%
Increased	93	58	35	37.6%
Ovarian all	127	78	49	38.6%

### ROC analysis

**Fig. 4:**
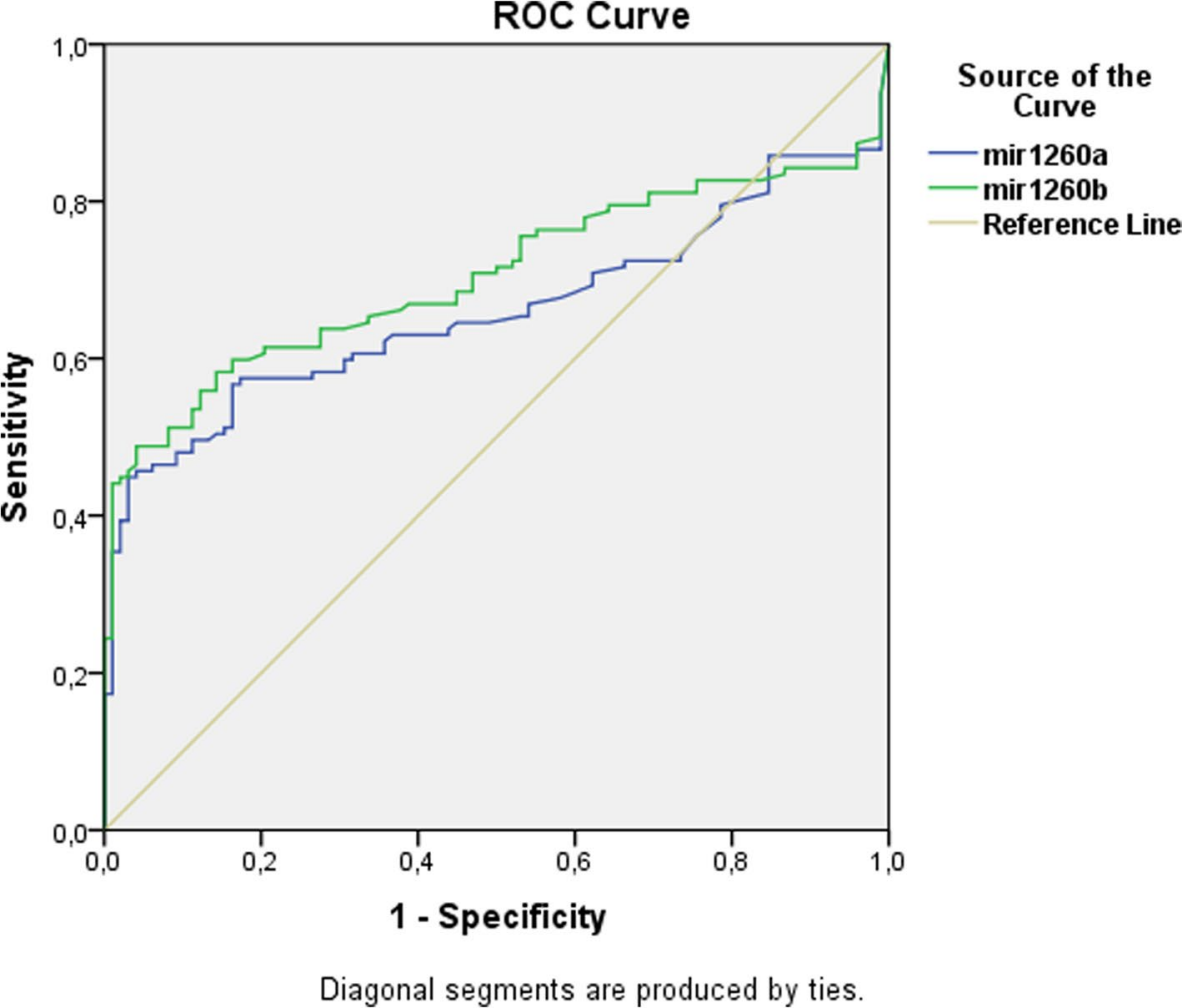
Graph of ROC-AUC values of miRNAs in separation of ovarian cancer patients and healthy groups. Blue line: miR-1260a; Green line: miR-1269b

We performed the Receiver Operator Characteristics (ROC) analysis for demonstrating the diagnostic performance of miR-1260a, and miR-1260b molecules as the candidate bioindicators- in other words their differentiation strength for ovarian cancer patient, and healthy control groups (Fig. 4). Table 5 demonstrates the ROC-AUC values, and 95% CI (Confidence Interval) results identified for each miRNA, and these results showed that the diagnostic strength of miR-1260a, and miR-1260b molecules were statistically significant in the diagnosis of ovarian cancer patients (p:0.000).

**Table 5:** ROC-AUC Values of miRNAs in the Separation of Ovarian Cancer Patients, and Healthy Groups

miRNAs	ROC-AUC	95% CI	p value
miR-1260a	0.660	0.588 - 0.733	.000
miR-1260b	0.704	0.635 - 0.773	.000

### Correlation between CA-125 value, and the expression level of miRNAs

We performed the “Spearman’s correlation analysis” for identifying whether there was a linear assocation between the numerical measurements of miR-1260a, and miR-1260b expression levels with the CA-125 values obtained from the clinical data of the ovarian cancer patients. There was no correlation, and no statistical significance between the miR-1260a, and miR-1260b expression levels with the Ca-125 numerical variables controlled in the beginning of treatment, and during treatment (p>0.05) (Table 6).

**Table 6:** Correlation Statistics Between miRNA Expression levels, and CA-125 Variables

p Values	CA-125 value on diagnosis	CA-125 value after treatment
**miR-1260a**	0.076	0.263
**miR-1260b**	0.094	0.122

### String analysis for identifying the Gene/Proteins that the miR-1260a, and miR-1260b were interacted

**Fig. 5:**
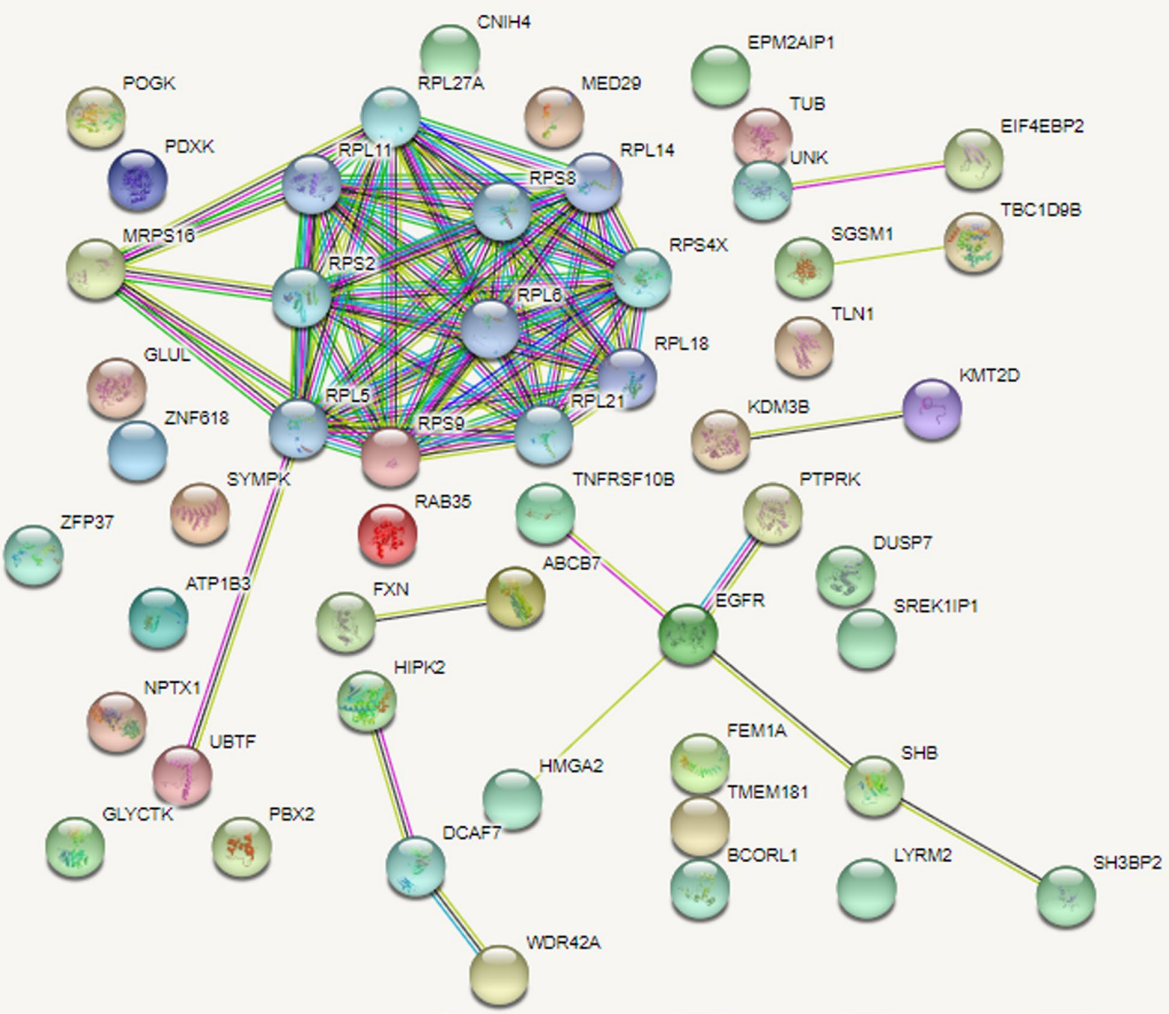
String analysis demonstrating the Gene/Protein interaction of miR-1260a

An experimental gene list was created for miR-1260a, and miR-1260b using the miRTarBase data bank. The estimated target genes for miR-1260a, and miR-1260b were identified using the TargetScan database. The cumulative gene targets smaller than zero for each miRNA were identified using the TargetScan. Accordingly, 4857 gene target for hsa-miR-1260a , and 388 gene targets for hsa-miR-1260b were detected. Then, the gene targets identified by TargetScan were compared with gene target lists identified by miRTarBase, and the common gene count for miR1260a was reduced to 42 Target Genes, and to 8 Target Genes for miR-1260b. The common gene targets detected for miR1260a by miRTarBase, and TargetScan were identified to have significant interactions in protein level after analysis performed using the String database (p:0.000). The results of the analysis, and the interacting genes are shown in Fig. 5.

The String Analysis showed that the most important protein family that the miR-1260a was associated was the ribosomal protein family which was known to be effective in the cellular stage of translation. Some studies in the literature investigated the effects of this protein family on the development of ovarian cancer [36, 37]. These studies showed that ribosomal proteins had significant, and critical functions in the development of ovary, and therefore may provide data about the pathophysiology, and treatment of ovarian dysfunctions.

**Fig. 6:**
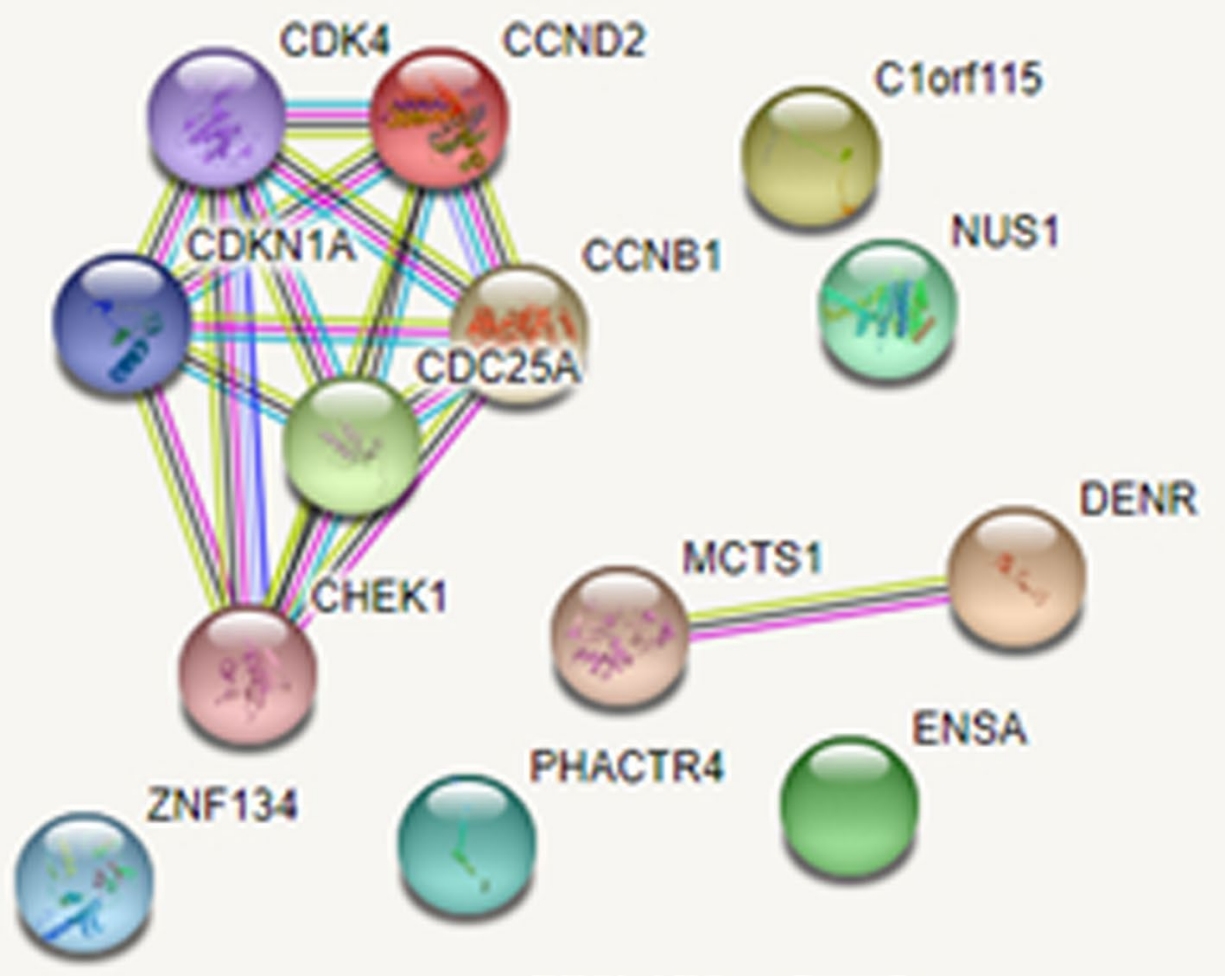
String analysis demonstrating the Gene/Protein interaction of miR-1260b

The common gene targets which were detected from miRTarBase, and TargetScan for miR1260b were found to have interaction in protein level after analysis performed using the String database (p:0.00361). The interacting genes are shown in Fig. 6.

The String analysis showed that the most important protein that miR-1260b was associated was the CHEK2 protein which was a member of the serine/threonine-protein kinase family. This protein has a significant role in inhibiting the control points in cell cycle, and in the activation of DNA repair. One another important protein was the CDK4 protein which was one of the members of cycline dependent kinase family. The abnormal activation of this protein has a role in the proliferation, and apoptosis of ovarian cancer cells. The cyclines functioning as the CDK kinase regulators (CCND2, CCNB1) were also striking. Cycline proteins were reported to be expressed in high levels in ovarian cancer tissues [7].

## Discussion

Approximately 22.240 individuals were diagnosed with ovarian cancer in 2018 in the USA. 14.070 of the diagnosed individuals were reported to have died of ovarian cancer [38]. 2018 Globocan data showed that 7 individuals in every 100.000 were diagnosed with ovarian cancer in Turkey. The age-adjusted highest incidence ratios were generally higher than 8 in 100.000 in developed regions including the North America, Central, and East Europe. The ratios were reported as 5.8 in 100.000 in South America, and as ≤3 in 100.000 in Asia, and Africa [5, 39]. Ovarian cancer is the most common gynecologic tumors with the highest mortality rate. The epidemiologic studies showed that hormonal, and reproductive factors were effective in cancer pathogenesis [40]. Ovarian carcinoma involves genetic, and epigenetic changes causing cell transformation. Although significant data has been obtained about the ovarian cancer oncogenes in the last decade, there is no clear data about the etiology. Various studies showed that miRNAs had significant role in various diseases including cancer, and stated that may be used as molecular biologic indicator and even as molecular target for cancer treatment or these miRNAs might be used as chemotherapeutic agent. Ovarian cancer needs to be investigated as the disease etiology is not clearly known, and there is no available biologic indicators for the use in the early diagnosis, and treatment of the disease. Therefore, it is important to search, and investigate for new biologic indciators to be described for this disease.

Many miRNA molecules associated with the development, diagnosis, and treatment of ovarian cancer have been investigated so far. The expression levels of miR-193b, miR-532, and miR-3064 were reported to be associated with the FIGO staging used in ovarian cancer staging, tumor diameter, histologic grade, ascitis accumulation, lymph node metastasis, and with the poor prognosis [41, 42]. However, miR-506 was shown to be associated with early FIGO Staging, and good prognosis and long time survival [43]. miR-199 was reported to have lower expression in ovarian cancer, and this miRNA inhibited the related gene pathway by blocking the activation of the c-MET, HIF1-alpha HIF2-beta, and IKK-beta proteins, and thus might be a therapeutic target [44–46]. miR-152 was shown to suppress the proliferation, and metastatis abilities of ovarian cancer cells by suppressing the ERBB3 expression in ovarian cancer cell strains. Therefore, miR-152 was reported as a possible significant molecular target in cell proliferation, invasion, and metastasis of ovarian cancer [47].

Different researchers reported various miRNAs which caused the development of resistance against ovarian cancer treatment [Jin, 2018 #570]. let-7 [48] , miR-9, miR-366, miR-424 [49], and miR-622 [50] were counted among these miRNAs. miRNAs circulating in the exosomal, and peripheral blood in the diagnosis and follow up of ovarian cancer were also reported. miR-21, miR-141, miR-200a, miR-200c, miR-200b, miR-203, miR-205, and miR-214 were found as exosomal in serum, and were correlated with the advanced stage ovarian cancer, and miR-106b, miR-126, miR-150, miR-17, miR-20a, and miR-92a found in plasma were shown to be possibly used in the differentiation of benign ovarian disease from malignant ovarian diseases [51]. The miR-92a, and miR-200b expression levels were detected to have differentiated in the ascitis fluid generated by ovarian cancer cells, and in the urines of patients compared with the healthy controls [52]. miR-630, and miR-205 expression differences in the invasion of the ovarian cancer cells were shown to be associated with the invasive behavior of the ovarian cancer patients [53]. miR-125 was found to be effective in miR-200 family, and miR-30d miRNA genes were effective in the epithelial mesenchymal transition (EMT) [54–57]. Researchers reported that miR-222-3p expression in ovarian cancer was associated with long time survival; the lower expression of miR-9, miR-595(81), and miR-15b with higher expression of WNT7a [58] were associated with shorter survival, and poor prognosis, in addition, the differences in the expression levels of miR-14 1[59], miR-200a, and miR-200c were reported to be associated with the progression-free survival (PFS). The PubMed screening until 2018 various above mentioned miRNAs were investigated in ovarian cancer, and were associated with ovarian cancer diagnosis, prognosis, survival, drug resistance, and metastatic characteristics.

There is no study in the literature investigating the role of miR-1260a, and miR-1260b miRNA molecules in ovarian cancer. The expression level of miR-1260a was investigated in breast, kidney , gastric cancer, malignant melanoma, hepatocellular carcinoma, colorectal carcinoma, and esophageal squamous cell carcinoma, and was detected to have been upregulated in these cancers [22–29]. In addition, miR-1260a expression level was reported to have high level expression in the serum, and FFPE tissues of prostate cancer patients compared with the BPH controls, and higher expression was also reported in prostate cell strains [30, 31, 60]. miR-1260a expression level was shown to have upregulated in prostate cancer patients compared with the BPH controls [60]. miR-1260a was reported as one of the 13 miRNAs with diagnostic biologic indicator property used in the classification of the FFPE of prostate cancer patients in Denmark population [30, 34].

The miR-1260b was also reported to have been investigated in various cancer types except ovarian cancer in the literature, and was associated with cancer, and showed abnormal expression. miR-1260b was reported to have been abnormally, and highly expressed in the dendritic cells in human peripheral blood, and therefore was suggested to be associated with immune response [33]. miR-1260b expression level was suggested to have increased in colorectal cancer tissues particularly with lymph node metastasis, and this was suggested to be associated with lymph node metastasis, and venous invasion, and showed the early stage metastasis. miR-1260b was suggested to be an important molecular biologic indicator in CRC prognosis [61]. In addition, miR-1260b was investigated in malignant melanoma patients, and miR-1260a was reported to show a different expression pattern in the surgical resection follow up of melanoma [62]. mi1260a expression was shown to have higher expression in atypical Spitz lesions compared with the levels in benign Spitz tumors [63]. mi1260b was detected to decrease the cell migration, and invasion in nonsmall cell lung cancer cell strains (A549). This results suggest that miR-1260b had an important role in inhibiting the metastasis in NSCLC patients, and might be a target molecule in NSCLC treatment [64].

Researchers showed that miR-1260b had extremely higher expression in renal cell cancer cells compared with the levels in normal kidney cells, and this expression was correlated with cell proliferation, invasion, and shorter survival, and miR-1260 showed this effect by inhibiting the Wnt signal pathway in these incidents [65].

The comparison of the ovarian cancer patients, and healthy control group with the analysis results in the present study showed that there was a highly significant difference (p:0.000). miR-1260a, and miR-1260b expressions were found to have increased between 16.1-45.08 fold in ovarian cancer patients compared with the healthy controls. The String analysis showed that miR-1260a was mostly associated with the ribosomal protein family effective in translation. There are some studies in the literature investigating the effects of this protein family of the development of ovarian cancer [36, 37]. The results of this studies showed that the ribosomal proteins had significant and critical functions in the development of ovarian cancer, and therefore was emphasized to provide information on the pathophysiology, and treatment of ovarian dysfunctions. The evaluation of all these data showed that the deteriorations developing in the ribosomal proteins of ovarian cancer might also be indirectly generated.

String Analysis performed for miR1260b showed that miR-1260b had a close association with ‘Serin/Treonin-protein kinase family member of CHEK2 protein, and cycline dependent kinase family members of CDK4 protein. One of which is effective in DNA repair, and the other is effective in cell proliferation, and apoptosis control suggesting that these genes contributed to tumor development by inhibiting the cell proliferation, and apoptosis in ovarian cancer development [7]. All these results may conclude that the interaction of 2 different gene families with miR1260 may cause the development of ovarian cancer. These results should be investigated in future studies.

## Conclusion

In summary, the significant expression of miR1260, and miR1260b in the peripheral blood lymphocytes of ovarian cancer patients compared with the healthy controls was first demonstrated by our group, the gene interactions of these two genes are suggested to have diagnostic significance owing to causing ovarian cancer. In addition, the present study suggests that miR1260a, and miR1260b molecules which were detected to have differentiated particularly in peripheral blood had prognostic significance, and therefore might be used as bioindicators. These molecules must also be investigated in the benign ovarian diseases for better understanding their importance in the early diagnosis, and diagnostic importance in ovarian cancer. In addition, the comparative investigation of the levels of these molecules in the peripheral blood during treatment is required to be studied for identifying their safety as prognostic biologic indicator.

## Data Availability

The datasets used and/or analyzed during the current study are available from the corresponding author on reasonable request.
